# Alcohol Use and Gamma-Glutamyltransferase Using a Mendelian Randomization Design in the Guangzhou Biobank Cohort Study

**DOI:** 10.1371/journal.pone.0137790

**Published:** 2015-09-10

**Authors:** Lin Xu, Chao Qiang Jiang, Kar Keung Cheng, Shiu Lun Ryan Au Yeung, Wei Sen Zhang, Tai Hing Lam, Catherine Mary Schooling

**Affiliations:** 1 School of Public Health, Li Ka Shing Faculty of Medicine, The University of Hong Kong, Hong Kong SAR, China; 2 Guangzhou No.12 Hospital, Guangzhou, 510620, China; 3 Public Health, Epidemiology and Biostatistics, University of Birmingham, Birmingham, UK; 4 School of Urban Public Health, Hunter College and CUNY School of Public Health, New York, New York, United States of America; Penn State College of Medicine, UNITED STATES

## Abstract

**Background:**

Observational studies and small intervention studies suggest alcohol raises gamma-glutamyltransferase (GGT). We used Mendelian randomization to assess the causal effect of alcohol use on GGT in older Chinese people.

**Methods:**

An instrumental variable (IV) analysis in 2,321 men and 2,757 women aged 50+ years from phase 3 of the Guangzhou Biobank Cohort Study with *ALDH2* (rs671) genotyped, alcohol use and GGT available was used to assess the causal effect of alcohol use on GGT. Rs671 was used as an IV and F-statistics was used to test for weak instrument hypothesis. An F-statistic of ≥10 indicates the IV is not weak.

**Results:**

In men, the F-statistic for rs671 on alcohol use was 70. Using IV analysis alcohol use increased GGT by 10.60 U/L per alcohol unit (10 gram ethanol) per day (95% confidence interval (CI) 6.58 to 14.62). The estimate was lower in observational multivariate regression: 3.48 U/L GGT per alcohol unit per day (95% CI 2.84 to 4.11) adjusted for age, education, physical activity and smoking. In women, rs671 was not associated with alcohol or GGT and the F-statistic was 7 precluding IV analysis.

**Conclusion:**

In Mendelian randomization, we found confirmative evidence that alcohol use increases GGT among Southern Chinese men. Moreover, we found that the ALDH2 variant rs671 was not associated with GGT among Southern Chinese women who generally consume very low levels of alcohol. Taken together our findings strongly suggest that alcohol increases GGT, although we cannot rule out the possibility that other unknown factors may cause a different relation between alcohol and GGT in other populations.

## Introduction

Gamma-glutamyltransferase (GGT) has been widely used as a marker for alcohol use in epidemiologic studies [1]. Observational studies have shown a positive association of alcohol use with blood levels of GGT [2–6]. However, observational studies are prone to biases from confounding, and may not be well suited to evaluating the causal effects of alcohol use. Randomized placebo controlled trials have shown that effective treatments for alcohol dependence or abuse which reduce alcohol use also reduce serum GGT concentrations [7, 8]. However, whether the reduction in GGT was mediated by the reduction in alcohol use or was due to other aspects of the treatments is unclear. Short-term intervention studies based on very small selected samples have shown that alcohol use increases GGT [9–11] but the results may not be applicable to the general population and cannot confirm the health effect of long-term alcohol use.

Given the limitations of the observational studies and ethical concerns regarding the carcinogenic effects precluding large scale randomized controlled trials of alcohol use, whether the association of alcohol use with GGT is causal or due to residual confounding remains to be determined. Mendelian randomization (MR) takes advantage of genetic variants present from conception and allocated randomly according to Mendel’s second law [12, 13]. Gene variants determining alcohol use can be used in instrumental variable analysis to elucidate the causal effects of alcohol on health [14]. However, a recent large MR study using functional polymorphisms in the alcohol dehydrogenase gene (*ADH1B*) as a genetic determinant of alcohol use did not show an effect of alcohol on GGT [15], probably because *ADH1B* is not only associated with alcohol use but also with the speed of alcohol metabolism [16, 17], which means that MR studies using *ADH1B* as a genetic instrument for alcohol use may give biased estimates of the effect of alcohol because effects may be due to genetic variation in *ADH1B* rather than to alcohol. Instead, a genetic marker, aldehyde dehydrogenase 2 (*ALDH2*) with single nucleotide polymorphism (SNP) rs671, has been shown by us to be a credible genetic instrument for alcohol use in southern Chinese men [14]. People with slow metabolization of acetaldehyde due to inactive *ALDH2* alleles may feel ill after drinking alcohol, and so in settings where alcohol use is discretionary tend to drink less. The *ALDH2* polymorphism rs671 has previously been used by us and others in MR studies to assess the effects of moderate alcohol use on cardiovascular disease risk factors and cognitive function [18–20].

We hypothesized that higher alcohol used would increase GGT, and the effect is due to alcohol but not the genetic variation in *ALDH2*. In the Guangzhou Biobank Cohort Study, taking advantage of this unique Southern Chinese setting in which alcohol use is generally low to moderate and may reflect genetic differences among men [14], we used *ALHD2* as a genetic instrument to obtain an unbiased estimate of the effect of alcohol use on GGT. We also assessed whether *ALHD2* alleles were associated with GGT in Southern Chinese women who rarely use alcohol.[21] A lack of association in such women would indicate that any effects seen in men are due to alcohol but not genetics. Finally, for comparison we assessed the associations of alcohol use with GGT using multivariable regression adjusted for confounders based on the same participants in an observational study design.

## Materials and Methods

### Participants

The details of the Guangzhou Biobank Cohort Study (GBCS) and MR and alcohol related studies have been reported elsewhere [14, 18, 19, 22]. Briefly, GBCS is a 3-way collaboration of Guangzhou 12th Hospital and the Universities of Hong Kong and Birmingham, UK. The GBCS baseline examination was conducted in three phases from 2003 to 2008, and participants were then followed up from 2008 to 2012. Participants were recruited from “The Guangzhou Health and Happiness Association for the Respectable Elders” (GHHARE), a community social and welfare organization. GHHARE is unofficially aligned with the municipal government. Membership is open to Guangzhou permanent residents aged 50 years or above for a nominal fee of 4 CNY (≈50 US cents) per month. GHHARE included about 7% of Guangzhou residents in this age group, with branches in all 10 districts of Guangzhou, the capital city of Guangdong province in southern China. The health examination included interview concerning lifestyle, family and personal medical history and assessment of anthropometric and clinical factors. Information on socioeconomic position and lifestyle including age, sex, education, smoking and alcohol use was collected by a computer based standardized questionnaire. Alcohol use was recorded in terms of frequency, type of beverage and usual amount per occasion. GGT was only measured in phase 3. The Guangzhou Medical Ethics Committee of the Chinese Medical Association approved the study and all participants gave written, informed consent before participation.

### DNA extraction and SNP analysis

Biological samples for DNA extraction used in the present study were obtained in GBCS phase 3 at baseline and in recruitment phases 1 and 2 at follow-up [14]. DNA was extracted at Guangzhou No. 12 Hospital either from fresh blood using a standard phenol-chloroform extraction procedure and stored at -80°C or from blood or buffy coat previously stored at -80°C using a standard magnetic bead extraction procedure. Genotyping of SNP rs671 to identify ALDH2 genotypes (AA, GA or GG) was performed using the MassARRAY system (Sequenom, San Diego, CA, USA) and the iPLEX assay at a commercial company (Beijing CapitalBio Corporation, Beijing, China). For logistic reasons genotyping for the three GBCS phases was performed at three different times, 953 men and 942 women had *ALDH2* genotyped in 2010, 2,748 men had *ALDH2* genotyped in 2011, then an additional 3,406 men and 1,535 women had *ALDH2* genotyped in 2012.

### Alcohol use

The exposure was continuous alcohol units (10 gram (g) ethanol) per day based on total alcohol consumption obtained from the frequency, quantity and type recorded at recruitment. Details of the assessment in alcohol use were reported previously [14, 23]. Specifically, we asked the participants how often they drank alcohol (once or twice per year, once every couple of months, <1 day/ week, 1–2 days/week, 3–4 days/week, 5–6 days/week, daily or almost every day), the type of alcohol usually consumed, and how much of each type of alcohol (beer, western grape wine, spirits, Chinese rice wine or Chinese spirits (high strength)) usually consumed per occasion, from which we calculated units per day. Participants who reported use of >30 alcohol units per day were considered as infeasible and were excluded [14]. Never drinkers were those who did not drink any alcoholic beverage throughout their life. Occasional drinkers were those who drank less than once per week, or drank only on special occasions, such as at a wedding party or festival, in the past 12 months. Moderate drinkers were people who drank at least once per week with less than or equal to 140 gram of ethanol for women and 210 gram of ethanol for men. Heavy drinkers were those who weekly drank more than 140 gram of ethanol in women and 210 gram of ethanol in men.[14] Former and occasional alcohol users were included as non-drinkers as reported previously [14]. We also conducted sensitivity analysis excluding former users and/or heavy users.

### Outcome

Serum GGT concentrations was the outcome. GGT were measured by Shimadzu CL-8000 Clinical Chemistry Analyzer (Shimadzu, Kyoto, Japan).

### Statistical analysis

We used linear regression to assess the strength of the association of ALDH2 variants (rs671) with alcohol units, from which we reported the F statistic and r^2^, to assess whether ALDH2 variants were associated with GGT and to assess whether the association was fully mediated by alcohol units. We used 2 stage least squares (2SLS) to estimate the possible causal effect of alcohol on GGT, i.e., the change in GGT per unit increase in alcohol intake per day. As a sensitivity analysis, we also used a 2-sample IV analysis in which the association between rs671 and alcohol use was estimated in participants with *ALDH2* genotyped and the association of rs671 with GGT was estimated in participants from phase 3. The ratio of the estimates and the confidence interval were calculated by seemingly unrelated regression and the Wald estimator [24] using the “suest” common in STATA. We did not adjust for confounders in the instrumental variable analysis because *ALDH2* genotypes randomly allocated at conception cannot be confounded by age or other environmental exposures. For comparison, we also present the associations of alcohol units with GGT under multivariable linear regression models adjusted for potential confounders including age, education, physical activity and smoking in the same participants. Few Southern Chinese women use alcohol [25]. *ALDH2* alleles correspond poorly with alcohol use among women, so we would expect rs671 to be a poor instrument for alcohol use among women [14]. Hence we present sex-stratified analysis. A two-sided significance level of α = 0.05 was used. All statistical analysis was done using STATA/IC 13.1 (Stata Corp LP, College Station, TX, USA).

## Results

Of 2,569 men and 7,519 women from phase 3 of GBCS, 2,321 (90%) men and 2,757 (37%) women with GGT and *ALDH2* genotyping were included in the analysis. An additional sample of 2,631 men from phases 1 and 2 with *ALDH2* genotyping but without GGT were included in a two-sample instrumental variable analysis.

About half of men (54%) and a minority of women (37%) were current alcohol users. Table 1 shows that in men *ALDH2* was strongly associated with alcohol use, such that men with two alleles for fast metabolization of acetaldehyde consumed 1.1 units of alcohol per day compared with 0.09 units in men with two alleles for slow metabolism of acetaldehyde. However, for women the association was much weaker because alcohol consumption was much less common. Rs671 was not associated with age, education, smoking or physical activity in men or women. ALHD2 was associated with GGT in men but not in women.

**Table 1:** Alcohol consumption and socio-demographic characteristics by *ALDH2* polymorphism rs671 in men and women from the Phase 3 of Guangzhou Biobank Cohort Study (2006–8).

	*ALDH2* polymorphism rs671			
	Two inactive alleles (AA)	One inactive allele (GA)	No inactive alleles (GG)	†P value
**Men**				
Number of participants	220	971	1,130	
Alcohol units, 10g ethanol per day, Geometric mean (95% CI)	0.55 (0.08–3.96)	1.28 (0.97–1.69)	2.49 (2.17–2.87)	<0.001
Gamma-glutamyltransferase, U/L, Geometric mean (95% CI)	25.0 (23.4–26.6)	26.1 (25.2–27)	30.2 (29.1–31.4)	<0.001
Age, years, Mean (SD)	63.3 (7.7)	63.8 (7.6)	63.4 (7.5)	0.40
Education, %				
Primary or below	25.5	30.4	27.6	0.45
Middle School	58.6	55.7	56.8	
College or above	15.9	13.9	15.6	
Smoking status, %				
Never	38.2	37.0	37.4	0.62
Former	29.6	25.5	26.8	
Current	32.3	37.5	35.8	
Physical activity, %				
Inactive	8.2	7.9	7.8	0.32
Moderate	28.6	33.5	35.8	
Active	63.2	58.6	56.4	
**Women**				
Number of participants	231	1,091	1,435	
Alcohol units, 10g ethanol per day, Geometric mean (95% CI)	0.31 (0.004–25.43)	0.36 (0.24–0.54)	0.48 (0.36–0.63)	0.22
Gamma-glutamyltransferase, U/L, Geometric mean (95% CI)	20.2 (19.0–21.4)	21.5 (20.8–22.2)	20.4 (19.8–21.0)	0.25
Age, years, Mean (SD)	59.9 (7.0)	60 (7.3)	60.1 (7.3)	0.91
Education, %				
Primary or below	42.4	41.7	40.4	0.62
Middle School	50.7	50.1	52.6	
College or above	6.9	8.3	7.0	
Smoking status, %				
Never	98.7	97.2	97.8	0.37
Former	0.9	0.9	1.1	
Current	0.4	1.9	1.2	
Physical activity, %				
Inactive	4.8	5.9	7.8	0.11
Moderate	27.7	24.5	22.7	
Active	67.5	69.7	69.5	

†P-value from analysis of variance (ANOVA) for continuous variables and from a χ^2^ test for categorical variables, 2 sided; Alcohol unit and GGT were log-transformed before the ANOVA analysis.

**Fig 1:**
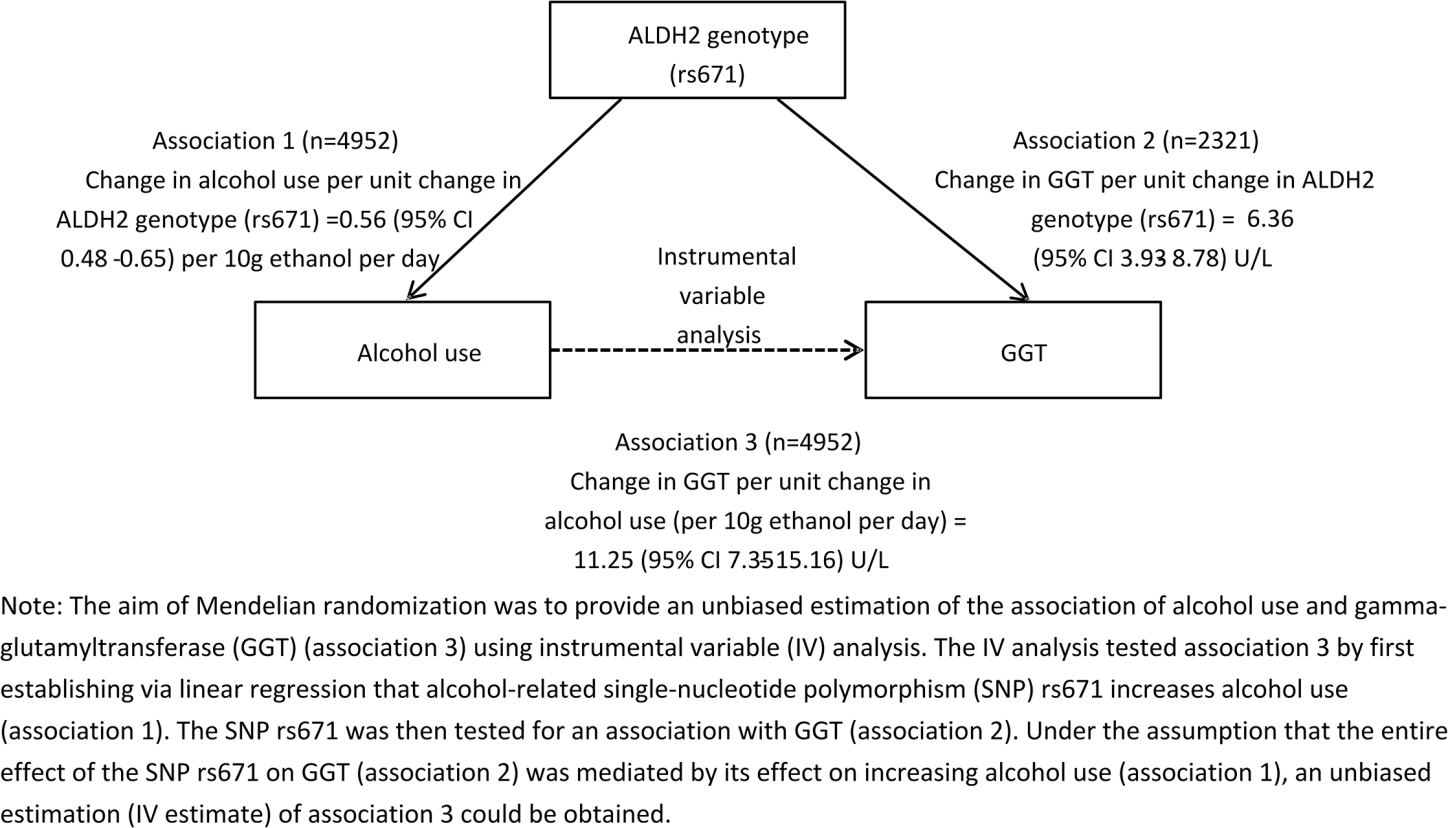
Two-sample Mendelian randomization of alcohol use and gamma-glutamyltransferase (GGT) in the Guangzhou Biobank Cohort Study.

The F-statistic for the association of rs671 with alcohol use was 70 in men, indicating that rs671 is a valid instrument for alcohol use in men. The r^2^ for rs671 on alcohol use was 0.03 in men. Table 2 shows that, in men, alcohol use increased GGT in instrumental variable analysis (10.60 U/L per alcohol unit, 95% confidence interval (CI) 6.58 to 14.62). After excluding heavy and former drinkers, the estimate became larger (45.89 U/L per alcohol unit, 95% CI 23.65 to 68.13) (Table 2). Moreover, the estimate was larger in men aged <65 years (15.66 U/L per alcohol unit, 95% CI 8.22 to 23.10) than those aged 65+years (5.89 U/L per alcohol unit, 95% CI 1.53 to 10.24). In two-sample instrumental variable analysis, alcohol use also increased GGT in men (11.25 U/L per alcohol unit, 95% CI 7.35 to 15.16) (Fig 1). After excluding heavy and/or former users, alcohol still increased GGT in men (Table 2). Observational multivariate regression also showed a positive association of alcohol with GGT in men (3.48 U/L per alcohol unit, 95% CI 2.84 to 4.11).

**Table 2:** Associations of alcohol (per 10g ethanol per day) with gamma-glutamyltransferase (GGT, U/L) using a Mendelian randomization design and an observational multivariable linear regression analysis among participants from the Phase 3 of Guangzhou Biobank Cohort Study (2006–2008).

		Mendelian randomization instrumental variable analysis			Observational multivariable regression analysis^†^	
	
Selection	Number	F-statistic	β	95% confidence interval	β	95% confidence interval
**Men**						
Total	2,321	70	10.60	6.58 to 14.62	3.48	2.84 to 4.11
Excluding heavy users	2,155	51	49.34	25.3 to 73.39	3.65	0.55 to 6.75
Excluding former users	2,166	73	10.15	6.33 to 13.97	3.51	2.87 to 4.15
Excluding heavy and former users	2,000	54	45.89	23.65 to 68.13	3.78	0.67 to 6.90
**Women**						
Total	2,757	6.8	-15.1	-75.61 to 45.42	0.99	-1.98 to 3.95
Excluding heavy users	2,748	5.4	-42.9	-220.17 to 134.36	11.15	3.51 to 18.80
Excluding former users	2,610	7.2	-9.35	-66.5 to 47.81	0.94	-2.04 to 3.93
Excluding heavy and former users	2,601	5.8	-25.8	-189.5 to 137.87	10.99	3.29 to 18.70

^†^ Adjusted for age, education, physical activity and smoking.

The F-statistic for the association of rs671 with alcohol use was 7 in women, indicating that rs671 is not a valid instrument for alcohol use in women in this population. The r^2^ for rs671 on alcohol use was 0.003. For completeness, Table 2 shows the instrumental variable and observational analysis for women.

## Discussion

Using IV analysis in a setting where alcohol use may reflect genetic differences in men [14], genetically higher alcohol use was associated with higher GGT. In contrast, among women where alcohol use is much less clearly a reflection of genetic differences, because of social pressure for Southern Chinese women to abstain from alcohol [25], these same genetic variants were not associated with GGT. Taken together these findings indicate convincingly that alcohol use increases GGT.

Using *ADH1B* as a determinant for alcohol use, a recent Mendelian randomization study did not show a significant effect of alcohol on GGT in alcohol drinkers even with a first-stage F-statistic of 59 [15], probably because *ADH1B* is not only associated with alcohol use but also with the speed of alcohol metabolism [16, 17], which means that *ADH1B* may not only be associated with GGT through alcohol use but *ADH1B* may also be directly associated with GGT through alcohol processing speed thereby giving a biased estimate of the effect of alcohol use on GGT. As such using *ADH1B* as a genetic instrument for alcohol use may violate the exclusion restriction assumption in Mendelian randomization, i.e., no link between the genetic instrument and the health outcome other than via the exposure. Moreover, weak instrument bias for *ADH1B* also cannot be completely ruled out because the high F-statistic was mainly driven by the very large sample size (n = 58,313) with a weak association of *ADH1B* with alcohol use (r^2^ = 0.001). Another recent Mendelian randomization study using *ADH1B* as genetic determinant for alcohol use also failed to show a significant effect of alcohol on high-density lipoprotein cholesterol [26], although the effect of alcohol on high-density lipoprotein cholesterol has been confirmed in the most up-to-date meta-analysis of experimental studies [27], supporting and suggesting *ADH1B* may not be an adequate instrumental variable for alcohol use. Instead, *ALDH2* has been shown to be an adequate and validated instrument (F statistic 75.1) for alcohol use in Southern Chinese men [14].

Using *ALDH2* as an instrumental variable in the current Mendelian randomization study, we found that the genetic effect of a unit change in alcohol use on GGT in men was larger than the association seen in observational linear regression. There are several possible explanations. First, observational study designs may be subject to residual confounding that biases associations toward the null. For example, hepatitis C virus (HCV) infection leads to higher GGT [28] and individuals with HCV infection tend to reduce alcohol consumption. Genetically determined alcohol use should not be confounded, especially by confounders which may mask the association of alcohol with GGT [29]. Second, the stronger association in Mendelian randomization after excluding heavy users could be due to canalization and developmental adaptation [30]. The genetic effect of alcohol on GGT may be modified via compensatory responses to environmental influences, i.e. up-regulating ALDH2 gene expression, to protect heavy alcohol users from increased GGT. This would also attenuate the association of genetically determined alcohol use with GGT. Third, the Mendelian randomization estimate for the effect of a unit change in alcohol use on GGT depends on the assumption that alcohol use is assessed without random measurement error, otherwise the association of genetic variants with alcohol use will be understated and correspondingly the instrumental variable estimate inflated [31]. Alcohol use is notoriously difficult to measure accurately, which may explain the higher estimate for the instrumental variable analysis than seen in observational studies [32]. Given the limited experimental data, it is difficult to know whether the effect of alcohol on GGT seen here from Mendelan randomization differs from what would be expected from an intervention. [10, 33]. Nevertheless, our findings suggest that the effect of alcohol on GGT is probably larger than that usually observed.

There were several limitations for our study. First, Mendelian randomization studies may be prone to confounding from linkage disequilibrium (LD) and the existence of pleiotropy. However, as *ALDH2* encodes mitochondrial *ALDH2*, the primary liver isoenzyme involved in the metabolism of acetaldehyde to acetate, *ALDH2* variants are unlikely to affect GGT independent of alcohol use, or be in LD with gene(s) affecting GGT [14]. Notably, *ALDH2* was not associated with GGT among the women in our sample who consume very little alcohol, suggesting *ALDH2* is only associated with GGT via alcohol use. Second, *ALDH2* mainly varies in East Asians. The effects of alcohol on health may vary with some as yet unknown differences between East Asians and other populations, although this is unlikely. Confounding by population stratification cannot be completely ruled out. However, our sample is restricted to permanent residents of one city in China, who are ethnically homogenous as it is difficult for internal migrants to obtain permanent residency. Our sample is not totally representative, however that would only create a bias if the association of *ALDH2* with alcohol use was different in our sample than in the general population. Third, alcohol use was self-reported, but we have previously validated self-reported alcohol use against high density lipoprotein-cholesterol.[34] Finally, given the low prevalence of alcohol use in Southern Chinese women, Mendelian randomization analysis of alcohol use, may be less optimal for women in this setting. However, causal effects should be consistent across sex, unless there is a biological reason, such as hormonal effects, for effects varying by sex.

## Conclusions

In conclusion, using Mendelian randomization, we found confirmative evidence for a direct causal effect of alcohol use on GGT. Our results underscore the value of Mendelian randomization to infer causality in observational epidemiology and to unravel underlying pathophysiologic mechanisms of alcohol-induced health effects.
